# Interrogating colorectal cancer metastasis to liver: a search for clinically viable compounds and mechanistic insights in colorectal cancer Patient Derived Organoids

**DOI:** 10.1186/s13046-023-02754-6

**Published:** 2023-07-17

**Authors:** Mario Cioce, Maria Rita Fumagalli, Sara Donzelli, Frauke Goeman, Valeria Canu, Daniela Rutigliano, Giulia Orlandi, Andrea Sacconi, Claudio Pulito, Alina Catalina Palcau, Maurizio Fanciulli, Aldo Morrone, Maria Grazia Diodoro, Marco Caricato, Anna Crescenzi, Martina Verri, Vito Michele Fazio, Stefano Zapperi, Massimo Levrero, Sabrina Strano, Gian Luca Grazi, Caterina La Porta, Giovanni Blandino

**Affiliations:** 1 Department of Medicine, Laboratory of Molecular Medicine and Biotechnology, University Campus Bio-Medico of Rome, Rome, Italy; 2 Institute of Translational Pharmacology, National Research Council of Italy (CNR), Rome, Italy; 3 Center for Complexity and Biosystems, Department of Environmental Science and Policy, University of Milan, Via Celoria 26, 20133 Milano, Italy; 4 CNR - Consiglio Nazionale Delle Ricerche, Biophysics Institute, Via De Marini 6, 16149 Genoa, Italy; 5 Translational Oncology Research Unit, Department of Research, Advanced Diagnostic and Technological Innovation, IRCCS Regina Elena National Cancer Institute, 00144 Rome, Italy; 6 Department of Research, Diagnosis and Innovative Technologies, UOSD SAFU, Translational Research Area, IRCCS Regina Elena National Cancer Institute, 00144 Rome, Italy; 7 Scientific Direction, IRCCS San Gallicano Dermatological Institute, Rome, Italy; 8 Clinical Trial Center, Biostatistics and Bioinformatics Unit, IRCCS Regina Elena National Cancer Institute, Rome, Italy; 9 Department of Pathology, IRCCS Regina Elena National Cancer Institute, Rome, Italy; 10 Colorectal Surgery Unit, Fondazione Policlinico Universitario Campus Bio-Medico, Università Campus Bio-Medico, Rome, Italy; 11 Unit of Endocrine Organs and Neuromuscular Pathology, Fondazione Policlinico Universitario Campus Bio-Medico, Rome, Italy; 12 Center for Complexity and Biosystems, Department of Physics, University of Milan, Via Celoria 16, 20133 Milano, Italy; 13 Istituto Di Chimica Della Materia Condensata E Di Tecnologie Per L’Energia, CNR - Consiglio Nazionale Delle Ricerche, Via R. Cozzi 53, 20125 Milano, Italy; 14 Cancer Research Center of Lyon (CRCL), UMR Inserm, CNRS 5286 Mixte CLB, Université de Lyon, 1 (UCBL1), 69003 Lyon, France; 15 Department of Experimental and Clinical Medicine, Hepato-Biliary Pancreatic Surgery, University of Florence, Florence, Italy; 16 CNR - Consiglio Nazionale Delle Ricerche, Istituto Di Biofisica, Via Celoria 26, 20133 Milano, Italy

**Keywords:** CRC, Liver metastases, Pentoxifylline, Dexketoprofen, Desloratadine, Organoids, 5-FU, CMAP, STAT3, IL-6, Chemoresistance

## Abstract

**Background:**

Approximately 20–50% of patients presenting with localized colorectal cancer progress to stage IV metastatic disease (mCRC) following initial treatment and this is a major prognostic determinant. Here, we have interrogated a heterogeneous set of primary colorectal cancer (CRC), liver CRC metastases and adjacent liver tissue to identify molecular determinants of the colon to liver spreading. Screening Food and Drug Administration (FDA) approved drugs for their ability to interfere with an identified colon to liver metastasis signature may help filling an unmet therapeutic need.

**Methods:**

RNA sequencing of primary colorectal cancer specimens vs adjacent liver tissue vs synchronous and asynchronous liver metastases. Pathways enrichment analyses. The Library of Integrated Network-based Cellular Signatures (LINCS)-based and Connectivity Map (CMAP)-mediated identification of FDA-approved compounds capable to interfere with a 22 gene signature from primary CRC and liver metastases. Testing the identified compounds on CRC-Patient Derived Organoid (PDO) cultures. Microscopy and Fluorescence Activated Cell Sorting (FACS) based analysis of the treated PDOs.

**Results:**

We have found that liver metastases acquire features of the adjacent liver tissue while partially losing those of the primary tumors they derived from. We have identified a 22-gene signature differentially expressed among primary tumors and metastases and validated in public databases. A pharmacogenomic screening for FDA-approved compounds capable of interfering with this signature has been performed. We have validated some of the identified representative compounds in CRC-Patient Derived Organoid cultures (PDOs) and found that pentoxyfilline and, to a minor extent, dexketoprofen and desloratadine, can variably interfere with number, size and viability of the CRC –PDOs in a patient-specific way. We explored the pentoxifylline mechanism of action and found that pentoxifylline treatment attenuated the 5-FU elicited increase of ALDHhigh cells by attenuating the IL-6 mediated STAT3 (tyr705) phosphorylation.

**Conclusions:**

Pentoxifylline synergizes with 5-Fluorouracil (5-FU) in attenuating organoid formation. It does so by interfering with an IL-6-STAT3 axis leading to the emergence of chemoresistant ALDHhigh cell subpopulations in 5-FU treated PDOs. A larger cohort of CRC-PDOs will be required to validate and expand on the findings of this proof-of-concept study.

**Supplementary Information:**

The online version contains supplementary material available at 10.1186/s13046-023-02754-6.

## Background

Colorectal cancer (CRC) is the third most commonly malignancy worldwide [1]. Surgical resection is the main therapeutic attempt, preceded by neoadjuvant chemo- and radio-therapy and followed by 5-fluorouracil (5-FU)-based adjuvant therapy for high stage cancer after resection [2]. For over 50 years, 5-FU has been a mainstay in the systemic treatment of colorectal cancer patients. However, approximately 20–50% of patients presenting with localized CRC progress to stage IV metastatic disease (mCRC) following initial treatment and the median overall survival of patients with mCRC is only 30 months [3]. Thus, interrogating CRC specimens to understand molecular determinants of such heterogeneity in the metastatic setting could help satisfy an unmet need.

Patient-derived tumor organoids (PDOs) are three-dimensional, self-assembling structures of cancer cells isolated from surgical specimens. PDOs were shown to recapitulate the cyto-architecture and, to a significant degree, the heterogeneity of the originating tumor [4]. PDOs can accurately represent the genomic landscape of their source, in terms of mutation rates, DNA methylation patterns, gene expression signatures and copy number variations (CNVs) [5]. This makes PDOs clinically relevant tools for disease modeling through predictive drug screening [6]. Aldehyde dehydrogenases (ALDH) are detoxifying enzymes that oxidize intracellular aldehydes thereby conferring resistance to alkylating agents [7]. The enzymatic activity of ALDH has been used to isolate stem-like cancer cell subpopulations and ALDH inhibitors reduced the viability of colorectal cancer cells [8]. We and others have shown in other tumor settings, that ALDHhigh cells do constitute a main chemoresistant cell subpopulation when challenged with antimetabolite or DNA damaging agents [9–11].

Interleukin 6 (IL-6) acts on cancer cells by inducing the expression of STAT3-dependent genes, thereby promoting cancer cell proliferation and survival [12, 13]. STAT3 itself may modulate IL-6 expression in a feedforward manner [14]. Of note, 5-FU induction of IL-6, TNFα, and IL-10 expression is an independent prognostic factors for OS in CRC [15]. Constitutive STAT3 activity was found to be abundant in CRC samples, but not in non-neoplastic colon epithelium [16] and implicated in the resistance to fluorouracil (5-FU)-based treatments [17, 18]. Finally, enriched nuclear localization of STAT3 was shown in ALDHhigh cells, suggesting a role for STAT3 signaling in the emergence and maintenance of these chemoresistant cells [19]. In this study, we have profiled matched and unmatched primary CRCs, liver metastasis and adjacent uninvolved liver tissue to get insights into the molecular determinants of the colon to liver metastatic progression. This effort has allowed us to capture additional aspects of the genomics of liver metastasis compared to primary tumors and adjacent liver, with metastases sitting in between the former and the latter tissues. We have conducted a Connectivity Map (CMAP) based screening for class of clinically validated compounds capable of interfering with a 22-gene signature that we identified as differentially expressed among primary tumors and liver metastatic lesions. We have tested four representative compounds on CRC PDOs and identified pentoxifylline as a potential agent towards CRC PDOs, alone or when co-administered with 5-FU. We elucidated the mechanism whereby pentoxifylline promotes 5-FU sensitivity and we found that this compound interfered with the -STAT3 mediated increase in chemoresistant ALDHhigh cells within the treated PDOs.

## Methods

### Samples

RNA-seq analysis was performed on 9 primary colon tumor samples, 25 samples derived from primary liver metastasis and 10 samples from the second or third wave of liver metastasis. All the primary tumor had at least one matched liver metastatic sample and for six of them RNA-sequencing was performed also on adjacent normal liver tissue (see Table S1).

### Reagents

Dexketoprofen, perphenazine desloratadine and pentoxifylline (Cayman Chemicals, Ann Arbor, Michigan, USA) were dissolved in DMSO. For all the experiments, the maximal concentration of DMSO used as a control was ≤ 0.05%. Recombinant IL-6 was from Peprotech (Cranbury, NJ, USA).

### RNA-seq analysis

Low-quality reads or reads containing adapter sequences were filtered using Trimgalore (v. 0.5.5) [20]. Trimmed sequences that aligned to human rRNAs or snoRNAs (May 2021) [21] using Bowtie (v. 1.2.2) [22] were discarded. Unmapped sequences were aligned to human genome (GRCh38 Primary Assembly) using STAR (v.STAR-2.7.1a) [23] with Ensembl gene annotation (release 99). The Sequence Alignment/Map format and SAMtools were used to mark duplicated sequences and Stringtie (v. 1.3.6) to estimate gene abundances. The same pipeline was used to analyze raw FASTQ data from two additional datasets obtained from ENA at EMBL-EBI (https://www.ebi.ac.uk) under the accession number PRJNA288518 and PRJNA603221 [24, 25]. The first dataset include quadruple-matched tissues (primary colorectal carcinomas, liver metastasis, normal adjacent liver and colon samples) from five patients while the second dataset include matched samples from primary colon cancer, liver metastasis and normal colon tissue from three patients. EdgeR was used for differential expression analysis filtering genes with low expression (> 10 counts in at least 10% of the samples). Multidimensional scaling (MDS) analysis was performed in R using plot MDS limma function (top = 2000). Differentially expressed (DE) genes were identified setting Benjamini–Hochberg adjusted p-value threshold to 0.01 and |logFC|> 4 using both glmFIT and glmQLfit functions. For dataset PRJNA288518, PRJNA603221 we consider as DE genes with |logFC|> 4 and *p*-value < 10^–3^ and *p*-value < 0.01 respectively.

Enrichment and gene ontology analysis was performed using Panther (v.16) and David (v. 6.8). List of tissue and cell line-enriched genes for intestine and liver was obtained from Protein Atlas database (Proteinatlas.org v.20.1).

Clustering and principal component analysis was performed at single pathway level using the R packages FactoMineR, Cluster, Factoextra and Pheatmap.

We considered a total of 151 pathways from Panther database (v. Feb. 2020–3.) for which human gene association was reported. For each dataset K-mean clustering was performed on those pathways with more than five genes expressed. For comparison between metastatic samples, the optimal number of clusters was found using clusGap R function. Briefly, clusGap function calculates the goodness of clustering measuring, for each number of clusters K_i_ = 1,..,K_max_, the distance of the within-cluster dispersion from the same quantity in a reference distribution. Maximizing the gap between these two gives the optimal (local) number of clusters K.

For the optimal K, K-mean clustering procedure was repeated 500 times over each pathway for each dataset and cluster composition is considered stable when is conserved in 95% of the replica. Library of Integrated Network-based Cellular Signatures (LINCS; http://www.lincsproject.org/) was interrogated using Connectivity Map (CMAP, version 1.1.1.43) [26] to identify compounds whose administration to cancer cells results in similar or opposite expression profile of our gene signature.

CMAP accepts as input a list of genes and generates a list of compounds rank-ordered, characterized by a score between -100 and + 100, based on the overlap between the query gene signature and the response after the perturbation in treated cells. The input lists should comprise at least 10 genes and at most 150 genes, labeled as up or downregulated. A large positive (negative) score associated to a compound suggest that it gives similar (opposite) signature compared to the input. For example, compounds with a score close to -100 are likely to decrease the expression of genes upregulated in the input signature. The scores can be calculated as an average response over the nine cell lines considered in CMAP or for one specific cell line.

We applied CMAP over two distinct signature of upregulated genes that characterize liver metastatic samples compared to either primary colon tumor or heathy adjacent liver tissue and considered the response in all the cell lines (global) and the specific effect in HEPG2 (liver hepatocellular carcinoma) and HT29 (colon adenocarcinoma). This allowed disentangling the contribution of cell lines from different tissues such as, for example, breast cancer or melanoma. In order to select the most relevant compounds for our analysis, we selected those with a score < -95 on all the cell lines (Global) and considering only HEPG2 or HT29.

### Flow cytometry

PDOs or freshly disaggregated tissue was mechanically and enzymatically disaggregated and filtered through a 70um filter mesh before staining. The following antibodies were employed, in separate tubes, each antibody matched to its isotype specific-related control antibody, in PBS1X-0.2% BSA, for 45 min at 4 °C, light protected. Aspecific staining from the isotype matched antibody was deemed as background and subtracted to the specific staining. For the Ki67 and CK20 staining, cell permebilization was performed before staining with the Cell Fixation & Cell Permeabilization Kit (ThermoFisher, Whaltam, MA, US). For viability assay, the disaggregated PDOs were stained with Sytox Blue Helix NP Blue (Biolegend, CA, US) for 5 min on ice before flow cytometry. Data were acquired with CytoFLEX Flow Cytometer (Beckman Coulter, IN, US) and analyzed with the provided companion software. The following antibodies were employed, all from Abcam (ABCAM, Cambridge, UK).

**Table:** 

FITC Anti-CD44 antibody [B-F24] (ab27285)
FITC Mouse IgG1 [B11/6]—Isotype Control (ab91356)
Alexa Fluor® 488 Anti-EpCAM antibody (ab237395)
Alexa Fluor® 488 Rabbit IgG, monoclonal—Isotype Control (ab199091)
Alexa Fluor® 488 Anti-Ki67 antibody (ab197234)
Alexa Fluor® 488 Rabbit IgG, monoclonal—Isotype Control (ab199091)
Recombinant PE Anti-Cytokeratin 20 antibody (ab209923)
PE Rabbit IgG, monoclonal [EPR25A]—Isotype Control (ab209478)
Anti-IL-6 antibody (ab6672)
Alexa Fluor® 647 Anti-CD130 (gp130) antibody (ab300159)
Alexa Fluor® 488 Anti-alpha smooth muscle Actin antibody (ab184675)

### PDO cultures

Patient Derived Organoids (PDOs) were obtained according to published protocols with no modifications [27]. Briefly, right colon adenocarcinoma biopsies were minced into < 1 mm pieces, and enzymatically and mechanically digested. Cells freed from tissue were filtered and suspended in extracellular matrix drops. Human organoid growing medium (hOGM) (Stem Cell technologies, Vancouver, CA) was added to the jellified drops. PDO cultures were passaged every 5-7dd by mechanical-enzymatic disaggregation as mentioned.

### Validation of PDO cultures

Flow cytometry was performed on both CRC specimens and passage 3 PDOs immediately after mechanical and enzymatic disaggregation and staining with for EpCAm, CK20, Ki67 and CD44.

### Cancer Associated Fibroblasts (CAF) isolation and propagation

Primary human colorectal cancer-associated fibroblasts were isolated from tumour tissues following published procedures [28] with some modifications. Briefly, minced and disaggregated CRC tissue was cultured in plastic dishes in 20% FBS containing hOGM for 72 h, to enrich for adherent cell subpopulations. After that, the growth medium was shifted to a 20% Fetal Bovine Serum (FBS) -containing advanced DMEM-F12 supplemented with non-essential-aminoacids (NEAA) (ThermoFisher, Waltham, MA, USA) and cells in suspension were removed at each passage by PBS 1X washing. Patient-derived CAFs samples were then tested for SMA expression and for negative EpCAM expression to evaluate epithelial cell contamination, before being used and within passage six from the isolation.

### *CAFs* + *PDO cocultures*

Disaggregated PDO-derived cells were mixed to a variable ratio (1:1 to 1:5 live cells) with CAFs and included into matrigel drops, as previously described, in complete hOGM, to start treatment 24 h later.

### PDO Treatment

PDO (or PDO + CAFs) were mechanically and enzymatically disaggregated to single cells and 500–1000 live cells were plated into 24-well plates 24 h before starting treatments. We classified the PDOs as resistant or sensitive based on an empirically defined response score (RS), according to the formula: number of formed organoids x average max diameter x viable cells (%) at time 0 day / number of organoids x average max diameter x viable cells (%) after 72 h. Please note that an RS score of 1 denotes no effect. Where indicated, the monoclonal IL-6 blocking antibody was from R&D Systems, Inc. Minneapolis, MN USA) and was used at a fixed concentration of 100 ng/ml. A monoclonal mouse IgG2B irrelevant antibody was used as a background control.

### RNA extraction and cDNA synthesis and gene expression

Total RNA was extracted using the RNAeasy minikit (QIAGEN). The first-strand cDNA was synthesized with the High Capacity RNA-to cDNA kit (ThermoFisher, Waltham, MA, USA). Gene expression was measured by real-time PCR using the SYBRGreen dye on a Step One instrument (ThermoFisher, Waltham, MA, USA). Specific primers for ALDH isoforms were described previously [9].

Gene Expression analysis of the 22 genes composing the colon to liver signature was performed at Eurofins Genomics (Milan, Italy) on a fee-for-service basis. Technical details will be available upon request.

### Detection of IL-6 by ELISA

The amount of IL-6 secreted in the medium of PDO cultures was quantified with Human IL-6 Quantikine ELISA Kit (R&D,Minneapolis, MN USA). PDO culture supernatants were centrifuged at 4C and diluted appropriately before detection.

### Detection of IL-6 by immunocytochemistry

Passage 3 organoids treated as indicated were cytospun on coated coverslips and fixed and permeabilized with sequential 10% neutral buffered formalin (NBF) and 100% methanol (on ice) before staining with anti- IL-6 antibody.

### Immunofluorescence staining

Cancer Associated Fibroblasts were plated in sterile Nunc Lab-Tek Chamber Slides (ThermoFisher, Waltham, MA, USA) and after 24 h fixed and permeabilized using a paraformaldehyde- methanol mix for 15 min. For blocking aspecific staining, a PBS1X/ 2%BSA solution was used*.*

### Aldehyde dehydrogenase activity (ALDH) detection

ALDH activity was assessed by flow cytometry with the ALDEFLUOR kit (Stem Cell Technologies Vancouver, BC, Canada) following the manufacturer’s instructions, as previously published [9, 11]. Briefly, the PDO-derived cells following disaggregation and filtering through a 70uM mesh, were incubated with BODIPY aminoacetaldehyde, which is converted into a fluorescent molecule (BODIPY aminoacetate) in the cytoplasm. Specificity of the fluorescence was shown using the specific ALDH inhibitor diethylaminobenzaldehyde (DEAB). To eliminate dead cells, cells were stained first with the viability stain Sytox-Red (Life Technologies Inc., Grand Island, NY, USA). Cell populations were identified using a using a Cytoflex flow cytometer (Beckman Coulter Life Sciences, IN, USA). The background fluorescence was subtracted from the specific one by using as reference the DEAB treated samples.

### Detection of Phospho-Stat3 (Tyr705) and pan-Stat3

For detecting the Phospho-Stat3 (Tyr705) and pan-Stat3, an ELISA based assay (Sigma-Aldrich, St. Louis, Missouri, USA), was used, according to the manufacturer’s instructions with the following modification: at least ten PDO-containing drops (with an average of 50 organoids/drop) were employed for each experimental point and the matrigel drops containing the PDOs were first mechanically disaggregated on ice, filtered and centrifuged (300 rpm for 5 min at 4C) before being resuspended in the ELISA kit lysis buffer at room temperature.

### Synergy calculation

Based on the raw data from the dose curve-effect on the organoid forming ability of the single- or co-administered compounds, we employed SynergyFinder [29] to calculate the Bliss Synergy Scores for the pentoxifylline (0-100uM) + 5-FU (0–12.5uM) treated PDOs. So when synergy score is lower than -10: the interaction between two drugs is likely to be antagonistic; -10 to 10: the interaction between two drugs is likely to be additive; > 10 the interaction between two drugs is likely to be synergistic [29].

We note that those concentrations were chosen based on the available PK data for both compounds as being the pharmacologically relevant ones [30–32].

### Statistics

GraphPad Prism (Version 9.0) was used to perform the data analysis. The data were from at least three independent experiments except where indicated and presented as mean ± standard deviation.

## Results

### Liver metastases resemble adjacent liver tissue closer than their matched primary tumors

We performed RNA-seq analysis on 9 primary colon tumor samples, 25 primary liver metastases and 10 samples from the second or third wave of liver metastatic lesions. All the primary tumors had at least one matched liver metastasis and for six of them RNA-seq was performed on adjacent normal liver tissue as well (Table S1).

**Fig. 1:**
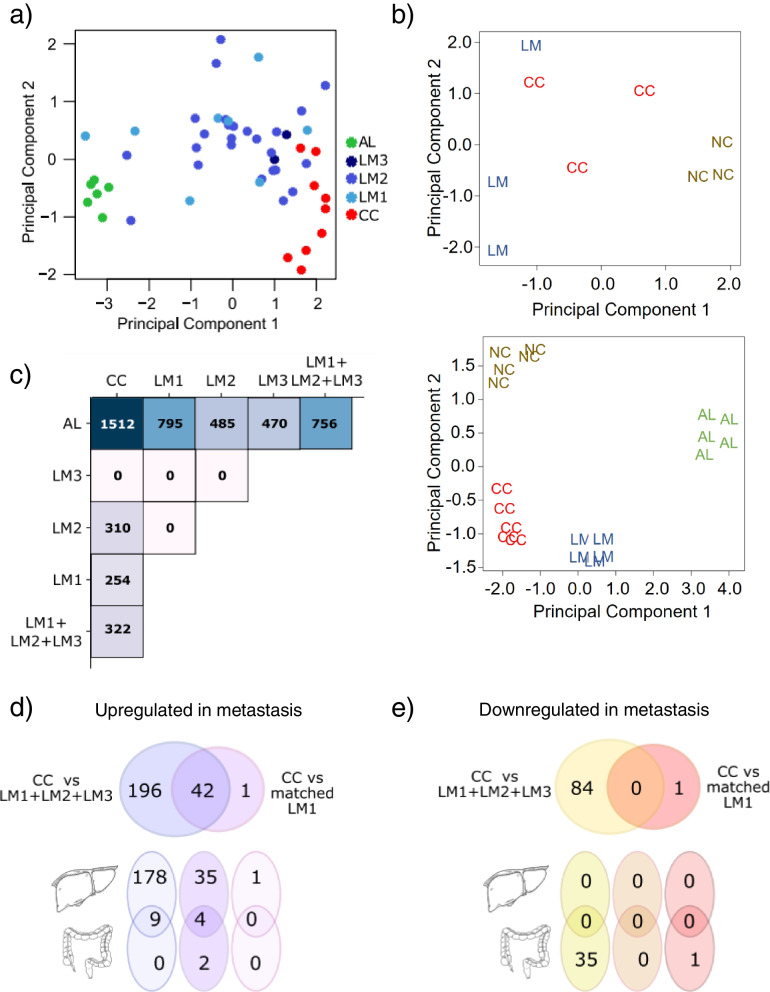
The colon to liver metastases are more similar to the adjacent liver tissue than the primary tumors. **a** Principal component analysis of all the samples in the study. (CC = Colon Cancer, red; LM1/LM2/LM3 = Colon-to-liver metastasis first/second/third wave, blue; AL = adjacent liver, green). **b**. Principal component analysis of two publicly available datasets of RNAseq of primary colon cancer (CC, red), liver metastases (LM, blue), adjacent liver (AL, green) and normal colon (NC, brown) samples. **c** Total number of differentially expressed genes between the various conditions (notation as in a). A large number of genes is deregulated between CC and AL, while it decreases in metastatic samples when considering each wave separately or merging metastatic samples together (LM1 + LM2 + LM3). **d**-**e** Number of up- and down-regulated genes in metastatic samples compared to primary tumors. We reported separately the number of DE genes for matched CC and LM1 samples (see Table S1) and over all the samples (left) as well as those in common between the two. The tables in the bottom report, for each subset, the number of genes annotated as liver-enriched, intestine enriched or both intestine and liver enriched according to Protein Atlas (see also Tab. S2-S3)

Principal component analysis (PCA) performed on all the samples, showed clustering of the data into three main groups: primary tumors, metastasis and adjacent normal liver samples (Fig. 1a). Notably, metastatic samples localized, in the PCA graph, between primary tumor and healthy liver samples, suggesting that the metastatic lesions were endowed with a higher level of similarity to the uninvolved tissue than primary tumors. We performed the same analysis on two publicly available datasets [24, 25] including matched primary and metastatic CRC samples as well as adjacent tissues. In both cases, PCA analysis provided similar results (Fig. 1b, upper and lower panels). To deepen this observation, we performed differential expression analysis between the mentioned subgroups of samples. Figure 1c reports the total number of DE genes between primary colon cancer (CC), adjacent liver (AL) and the different waves of liver metastasis (LM1, LM2, LM3) or all the metastatic samples without distinguishing among different waves of metastasis. In detail, when focusing on adjacent liver samples, the number of genes DE against primary CRC samples (1512) was halved (756) when we compared adjacent liver to the complete set of metastatic samples. This result was coherent with our previous observations from the PCA. However, the amount of DE genes between primary CRC tumor and corresponding liver metastases was even smaller considering both the metastatic waves separately (254/310/0) or as a whole (322) (Fig. 1c), suggesting that metastatic samples still retained characteristics of the tumor they originate from. The lack of significantly DE genes between LM3 and other tumor samples may be related to the small sample size of this specific subgroup.

Next, we analyzed separately the genes overexpressed and downregulated, focusing on matched primary CRC and metastases.

Considering the subset of 322 DE genes between all primary CRC and all the metastatic samples we identified 238 upregulated and 84 downregulated genes (Fig. 1d,e). The same analysis performed only on the subset of eight primary colon tumor tissues and their corresponding first round liver metastasis (CC – LM1 matched) revealed a core of 43 genes upregulated in metastatic samples and only one gene downregulated (Fig. 1d,e). The Venn diagram in 1d shows that a large fraction (42/43) of the genes upregulated in metastatic samples was conserved in both matched and unmatched analysis. Among these, 35 genes were reported as characteristic of liver healthy tissue (liver-enriched) in the Protein Atlas but not intestine-enriched; four genes resulted both liver and intestine-enriched while only two genes were annotated as specifically intestine-enriched (Fig. 1d, bottom) and one gene did not result as tissue-specific.

This signature was even stronger when considering the whole set of overexpressed genes in unmatched specimens, with essentially all of them (226/228) annotated as liver-enriched or expressed in both liver and colon according to Protein Atlas (Fig. 1d, bottom). On the other hand, we did not find a core of DE genes downregulated in metastatic matched and unmatched samples and less than half of all the genes downregulated (35/85) were characteristic of the intestine, while none was annotated as liver-enriched (Fig. 1e, bottom). Pathway enrichment analysis confirmed that the core of the upregulated genes was involved in liver-related processes such as blood coagulation, plasminogen activation and nicotin degradation (Table S2). We verified whether the identified DE genes could be detected in independent datasets retrieved from public databases. In detail, the comparison between primary and metastatic samples from two analyzed independent datasets [24, 25] resulted in 65 genes upregulated in metastasis versus primary CRCs and common to both the public datasets and to our samples (Table S3). 22 out of the 65 genes were included within the more stringent core of the 43 DE genes derived by the intersection of fully matched samples (Table 1 and Table S3). Thus, despite including unmatched samples and thereby increasing the number of DE and the general background noise of the analysis, a significant closer proximity of the liver metastasis to the liver tissue was still recorded (as compared to the primary CRC).

**Table 1:** List of the 22 genes upregulated in colon to liver metastasis compared to primary colon tumor. Data represent the intersection between our original dataset and two publicly available ones, as indicated in the text

Gene	Gene name	Ensembl ID	RNA tissue enrichment
**APOB**	Apolipoprotein B	ENSG00000084674	intestine: 224.8;liver: 442.2
**APOF**	Apolipoprotein F	ENSG00000175336	liver: 114.0
**ASGR2**	Asialoglycoprotein receptor 2	ENSG00000161944	liver: 154.1
**C9**	Complement C9	ENSG00000113600	liver: 419.5
**CPB2**	Carboxypeptidase B2	ENSG00000080618	liver: 246.8
**CPN2**	Carboxypeptidase N subunit 2	ENSG00000178772	liver: 225.5
**CRP**	C-reactive protein	ENSG00000132693	liver: 1139.7
**CYP2E1**	Cytochrome P450 family 2 subfamily E member 1	ENSG00000130649	liver: 802.4
**DPYS**	Dihydropyrimidinase	ENSG00000147647	kidney: 42.2;liver: 103.6
**FGA**	Fibrinogen alpha chain	ENSG00000171560	liver: 2009.3
**FGB**	Fibrinogen beta chain	ENSG00000171564	liver: 1908.6
**FGG**	Fibrinogen gamma chain	ENSG00000171557	liver: 2225.7
**FGL1**	Fibrinogen like 1	ENSG00000104760	liver: 450.7
**GC**	GC, vitamin D binding protein	ENSG00000145321	liver: 552.1
**HPR**	Haptoglobin-related protein	ENSG00000261701	liver: 151.4
**ITIH3**	Inter-alpha-trypsin inhibitor heavy chain 3	ENSG00000162267	liver: 326.9
**ITIH4**	Inter-alpha-trypsin inhibitor heavy chain family member 4	ENSG00000055955	liver: 461.4
**LBP**	Lipopolysaccharide binding protein	ENSG00000129988	liver: 429.7
**ORM1**	Orosomucoid 1	ENSG00000229314	liver: 834.6
**PRG4**	Proteoglycan 4	ENSG00000116690	adipose tissue: 20.9;liver: 76.0
**VTN**	Vitronectin	ENSG00000109072	liver: 321.7
**SLC13A5**	Solute carrier family 13 member 5	ENSG00000141485	liver: 150.4;salivary gland: 153.9

Altogether, our analysis suggests that metastases exhibited an hybrid phenotype acquiring some characteristics and processes common to the surrounding, unaffected liver tissue while losing, at the same time, some features of the originating colon tumor, in agreement with the earlier PCA distribution data (Fig. 1a,b).

### Further characterization of liver metastases

We performed k-mean clustering of the samples based on their gene expression profile at the pathway level. When considering the 50 samples in our dataset we found that this classification in clusters resulted stable in 15 different pathways (Figure S1). We could identify three main blocks, roughly corresponding to primary tumor, liver metastasis, and adjacent liver samples (Figure S1). Interestingly, a small group of four metastatic samples clustered preferably (> 10/14) with liver tissue and only one sample clustered with primary tumor. This provided further support to the similarity of metastatic tissue to the liver tissue already mentioned (Figure S1).

### Identifying drugs to interfere with the colon to liver metastasis signature

**Fig. 2:**
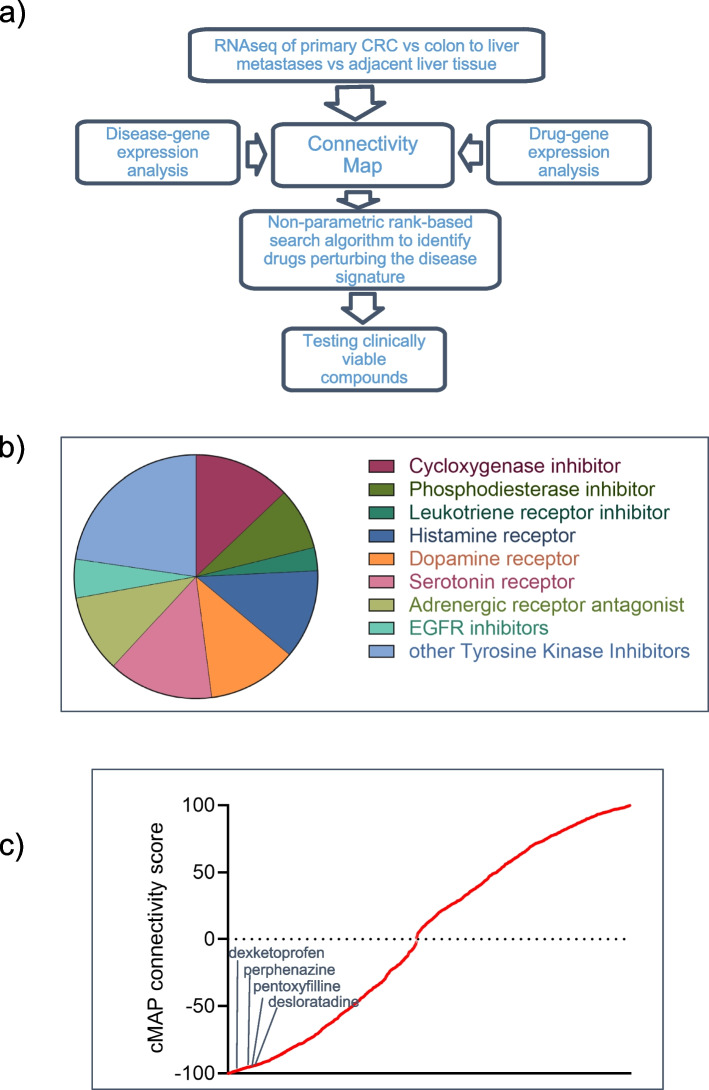
Identification of clinically viable compounds. **a** Schematic workflow of the in silico search for clinically viable drugs based on the identified DE genes. Briefly, the colon to metastasis disease gene signature (Table 1) was used to query the CMAP database, a collection of paired gene expression profiles from ctrl- and drug-treated cell lines. A positive correlation denotes the degree of similarity and a negative correlation emphasizes an inverse similarity between the query signature and the reference profile generated by the chemical perturbation. **b** Pie chart showing the distribution of the most represented class of compounds identified among those more negatively correlated with our bait signature (connectivity score between -100 and -50). **c** Representative histogram showing all the identified compounds ranked by the CMAP connectivity score. On y-axis is reported the connectivity score with negative values indicating negative-correlation and positive value indicating positive correlation. Lines indicate the approximate position of the four compounds selected for further testing

Next, we conducted a pharmacogenomic screening for compounds capable of interfering with the identified 22-gene signature (Table 1) which, as before mentioned, was found to differentiate primary CRC from matched liver metastases and was validated in a large public database (Fig. 2a). To do this, the Library of Integrated Network-based Cellular Signatures (LINCS; http://www.lincsproject.org/) was interrogated using Connectivity Map (CMAP, version 1.1.1.43; https://clue.io/) to identify compounds whose administration to cancer cells resulted in similar or opposite expression profile of the mentioned gene signature [33]. We applied CMAP to the distinct signatures of 21 annotate genes (SLC13A5 was left out because not annotated in CMAP) (Fig. 2a). The class of drugs that were potentially capable to revert the detected signature included cyclooxygenase (COX) and leukotriene receptor inhibitors, dopamine receptor antagonists, histamine receptor antagonists, adrenergic receptor inhibitors, serotonin receptor antagonists, phosphodiesterase inhibitors, adrenergic receptor antagonist, EGFR inhibitors and other Tyrosine Kinase inhibitors (TKI) (Fig. 2b and Table S4). The working hypothesis beyond our approach was that compounds capable of interfering with such a gene signature may be capable of attenuating protumorigenic features of the primary tumor. We choose, among the candidate drugs resulting from this in silico approach, dexketoprofen, perphenazine, desloratadine, pentoxyfilline; each one of these as being representative of an enriched class of compound and negatively correlated with the 22-gene signature, endowed with a known safety profile and a literature-based support toward anticancer activity (see Discussion please) (Fig. 2c).

### Validating the model in Patient-Derived-Organoids (PDOs)

To validate our findings in a clinically relevant model system, we tested some of the identified drugs in early passage CRC PDOs established in our lab. Patient derived organoids are deemed to recapitulate, in a clinically meaningful way, the main features of the originating tumor specimen [34]. We established representative PDOs obtained from four CRC patients by following slightly modified procedures published elsewhere [4]. All the specimens exhibited an adenocarcinoma histotype, were derived from right colon lesions and variable clinico-pathological features (Fig. 2a).

**Fig. 3:**
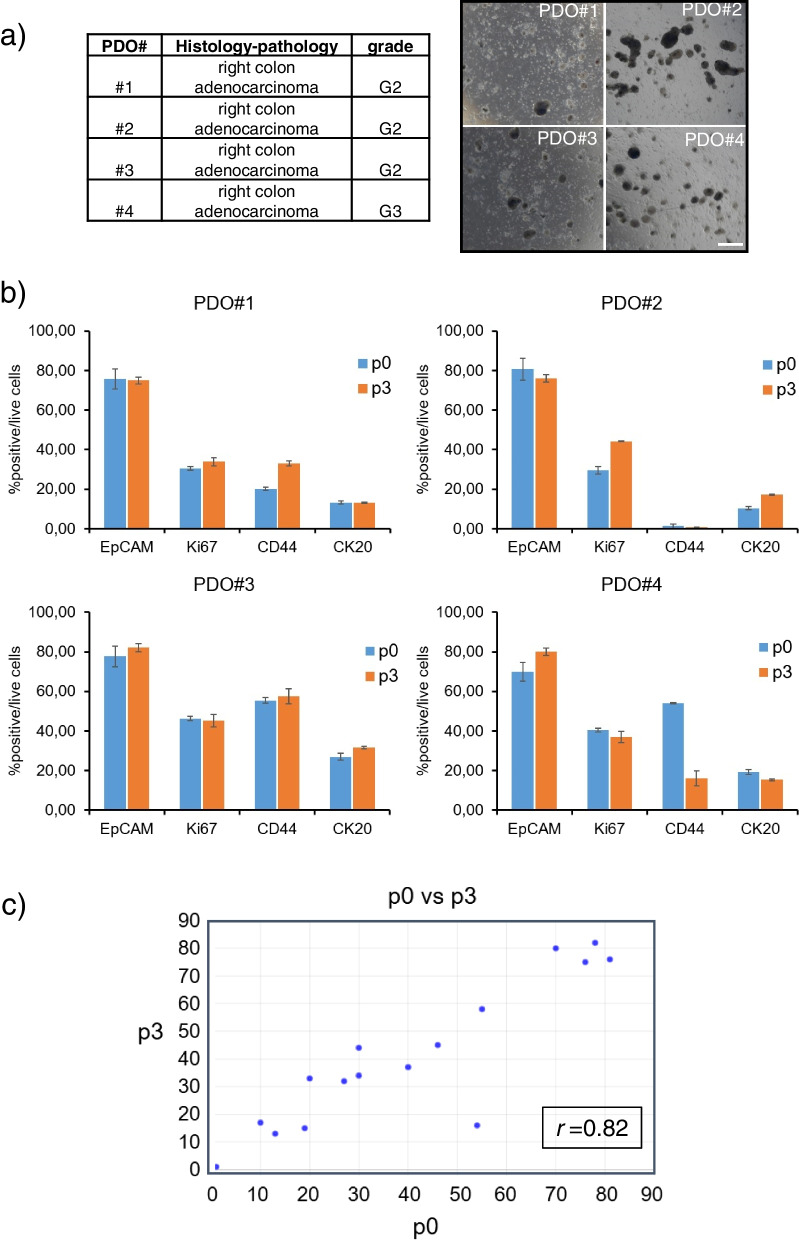
Characteristics of the CRC-derived PDOs. Patient-Derived-Organoids were obtained from four right colon adenocarcinoma specimens as described in methods. **a** Right panel: Clinico-pathological features of the obtained specimens. Left panel: representative micrographs of the four PDO cultures obtained from the specimens indicated in 3a, left. Size bar: 200 µm. **b** Validation of the PDO cultures. Upper panel: histograms showing the percentage of cells positive for EpCAM, Ki67, CD44 and CK20 in the CRC tissue immediately after the mechanical disaggregation (passage 0, p0). Lower panel: histograms showing the percentage of cells positive for the expression of the above antigens in PDO cultures disaggregated after three sequential passages (passage 3, p3). **c** High correlation between the number of positive cells within the p0) and the p3) specimens was shown (r = 0.8213, *p* < 0.01) suggesting a similar composition in cell subpopulations between the p0 and the p3 PDOs

We first proved that primary CRC specimens and passaged PDOs were cytologically similar. Indeed, the obtained PDOs, after serial passaging (passage 3) expressed a repertoire of intracellular and membrane markers closely similar to that of the originating specimen (freshly disaggregated, passage 0) (Fig. 2b and Figure S2). In detail, high correlation was shown between the expression of Ki67, EpCAM, CD44, CK20 in the specimen immediately after disaggregation at passage 0, and the percentage of positive cells in the disaggregated PDOs at passage 3 (r = 0.82; Fig. 3c). Notably, the ratio between the percentage of cells expressing each antigen was conserved among the two group of samples (Fig. 3c), suggesting that the overall distribution of the cell subpopulations was conserved. We noticed a trend toward an increased expression of EpCAM in the passaged PDOs (Fig. 3b), which may reflect the selection, during PDO culturing, for epithelial components [35].

**Fig. 4:**
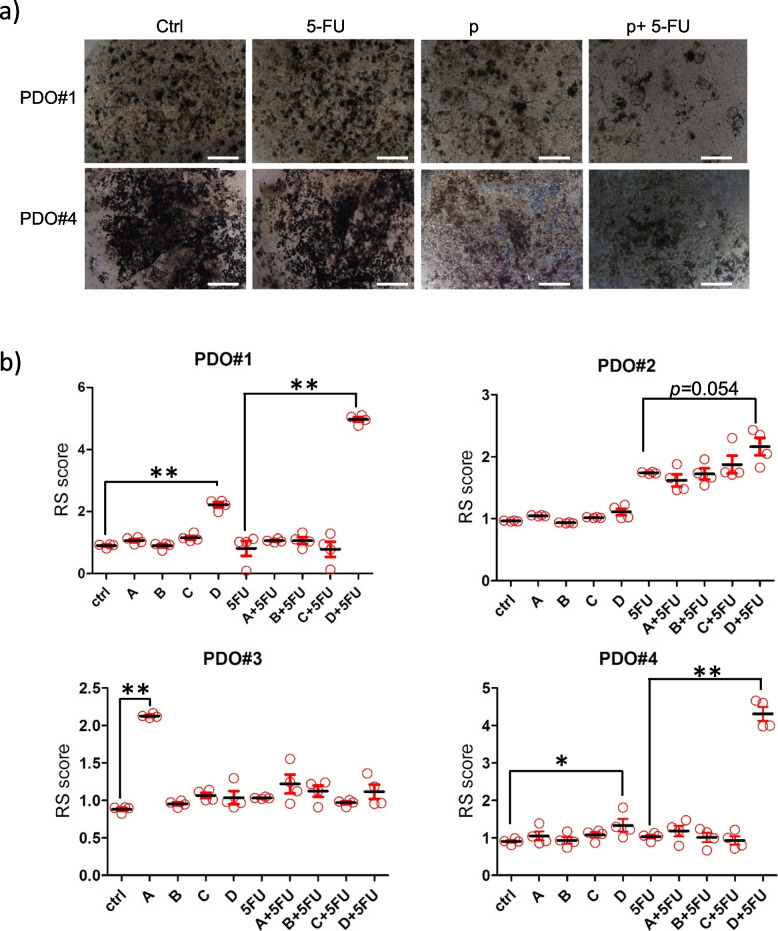
Validation of the in silico screening with CRC PDOs. **a** Representative micrographs of two PDO cultures treated with 0.05% DMSO (ctrl)- or pentoxifylline(p) and subsequently with saline-(ctrl) or 5-FU. Micrographs taken at 72 h after treatment started. Scale bar: 200 µm. **b** Graphs showing the RS score for the PDOs treated with ctrl (DMSO 0.05%) or with the compound A (dexketoprofen), B (perphenazine), C (desloratadine) or D (pentoxifylline) and challenged 6.5 h later with ctrl (saline) or 5FU. The RS score was obtained according to the following formula: number of formed organoids x average max diameter at time 0 day / number of organoids x average max diameter after 72 h, as described in the methods section. Statistics: * *p* < 0.05; ** *p* < 0.01; no asterisk: not significant

For the treatments, the four PDOs at passage 2 were mechanically and enzymatically disaggregated and 24 h later treated with literature-derived doses of 5-FU and of the chosen compounds (see methods please). We classified the PDOs as resistant or sensitive based on an empirically defined response score (RS), described in the methods section (Fig. 4a,b). The treatment with 5-FU alone revealed that in PDO#1, PDO#3 and PDO#4 the number and size of the formed organoids were not significantly affected by pharmacologically relevant doses of 5-FU (6.25uM-72 h) [36], while the PDO#2 showed sensitivity to 5-FU (Fig. 4b and Figure S3). The drugs selected from the identified list (Fig. 2c and Table S4), namely dexketoprofen, perphenazine, desloratadine, pentoxifylline, were added at 50uM, 20uM, 10uM, 20uM, respectively, to test their activity in the presence of 5-FU or ctrl (saline). 5-FU was added 6.5 h after the addition of the selected compounds. When evaluating the response of the formed organoids to the compounds as single agents or combined with 5-FU, we found that this was rather heterogeneous (Fig. 4b). As discussed later, this may be quite expected when using clinically relevant models which closely reflects the inter- and intra-patient tumor heterogeneity. Based on the response score (RS), we found that, while some PDOs were sensitive to the compound administered as a single agent, others exhibited sensitivity only in combined 5-FU treatment. In detail, PDO#1 was sensitive to pentoxifylline when both single- or co-administered. PDO#3 exhibited sensitivity to dexketoprofen as a single agent while for PDO#2 no synergistic effects of the compounds were recorded, possibly because of the prominent sensitivity to 5-FU. Still, we could observe a reproducible trend toward an increased RS score when pentoxifylline was co-administered with 5-FU (*p* = 0,054). Finally, PDO #4 was sensitive only to the combined treatment with 5-FU and pentoxifylline (Fig. 4b).

**Fig. 5:**
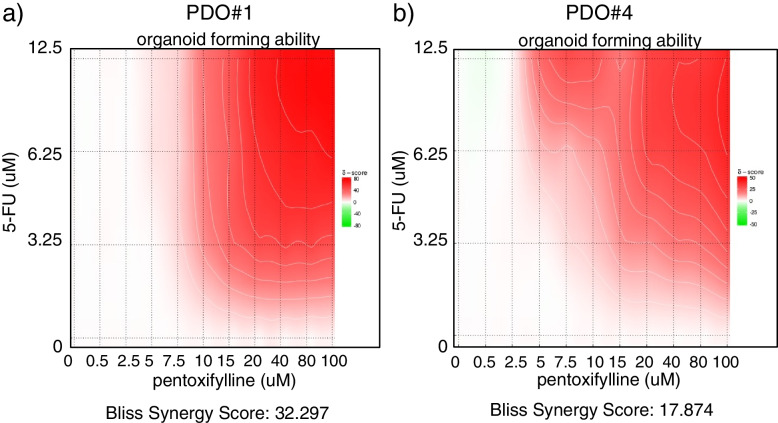
Pentoxifylline exhibited synergistic activity with 5-FU toward the Organoid Forming Ability. Representative graphs of the effect of the combined pentoxifylline and 5-FU treatment on the organoid forming ability of PDO#1 (**a**) and PDO#4 (**b**). The Bliss synergy score calculated was > 32 and > 17 for PDO#1 and PDO#4, respectively, indicating synergistic effect of the two administered compounds. Details are available in the methods section

Overall, our analysis revealed that, despite a high degree of variability, three out of four PDO cultures were sensitive to pentoxifylline (notably a trending effect was recorded for PDO#2), either as a single agent (PDO#1) or combined to 5-FU (PDO#1, PDO#2, PDO#4). Next, we aimed at studying the chemosensitizing effect of pentoxifylline toward 5-FU in the PDO#1 and PDO#4, which exhibited the highest sensitivity to pentoxifylline treatment (Fig. 5).

### The interaction of pentoxifylline and 5-FU is synergistic

To detail the interaction between pentoxifylline and 5-FU we treated the PDO#1 and PDO#4 with pentoxifylline (0-100uM) and 5-FU (0–12.5uM), a range of doses based on the available PK data for both compounds [30–32]. Pentoxifylline was, dose dependently, capable of potentiating the effect of 5-FU on the Organoid Forming Ability of the treated cells (Fig. 5). When calculating the Bliss synergy score [29] (BS), we found their interaction to be highly synergistic, with BS of > 17 and > 34, for PDO#1 and PDI#4, respectively (Fig. 5a,b).

### Pentoxifylline treatment blunted the 5-FU-mediated increase of chemoresistant ALDHhigh cells

**Fig. 6:**
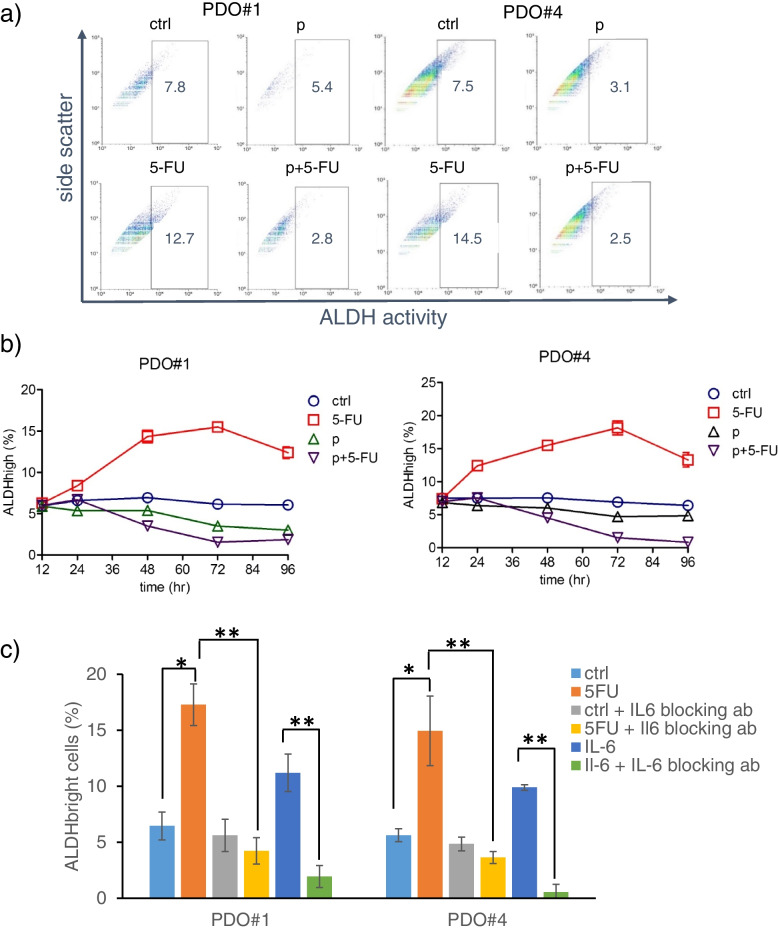
Pentoxifylline blunted the 5-FU-mediated increase of ALDHhigh cells in PDOs (**a**) 48 h after the indicated treatments, freshly disaggregated and filtered PDO-derived cells were analyzed for their ALDH activity by means of the ALDEFLUOR assay as described in the methods. Representative dot plots. **b** Graphs showing the percentage of ALDHhigh cells from quadruplicate experiments during a (12-96 h) time course. **c** A 5-FU- stimulated increase of IL-6 mediated the emergence of ALDHhigh cells. Representative histograms showing the levels of ALDHhigh cells in the PDO#1 and PDO#4 pretreated with a mock- or a IL-6-neutralizing antibody (100 ng/ml, 1 h) before being challenged with ctrl- or 5-FU as in (**a**). ELISA assay. Statistics: * *p* < 0.05; ** p < 0.01; ns: not significant

Those endowed with high levels of Aldehyde dehydrogenase represent a cell subpopulation that we and others have shown to mediate resistance to therapy in various settings [8–11, 19, 37, 38]. Given the effect of pentoxifylline toward 5-FU sensitivity (Figs. 4 and 5), we investigated whether this latter involved a rearrangement of ALDHhigh cells. Flow cytometry analysis of the PDO#1 and PDO#4 treated with 5-FU revealed a more than two folds increase of ALDHhigh cells as compared to ctrl- treated PDOs (Fig. 6a). The increase of ALDHhigh cells was time dependent, reaching a plateau after 72 h (Fig. 6b). Pentoxifylline, alone or with 5-FU, significantly attenuated such ALDHhigh cell increase (*p* < 0.05) (Fig. 6a,b).

### IL-6 released after 5-FU treatment of PDOs may mediate the increase of the ALDHhigh cells

**Fig. 7:**
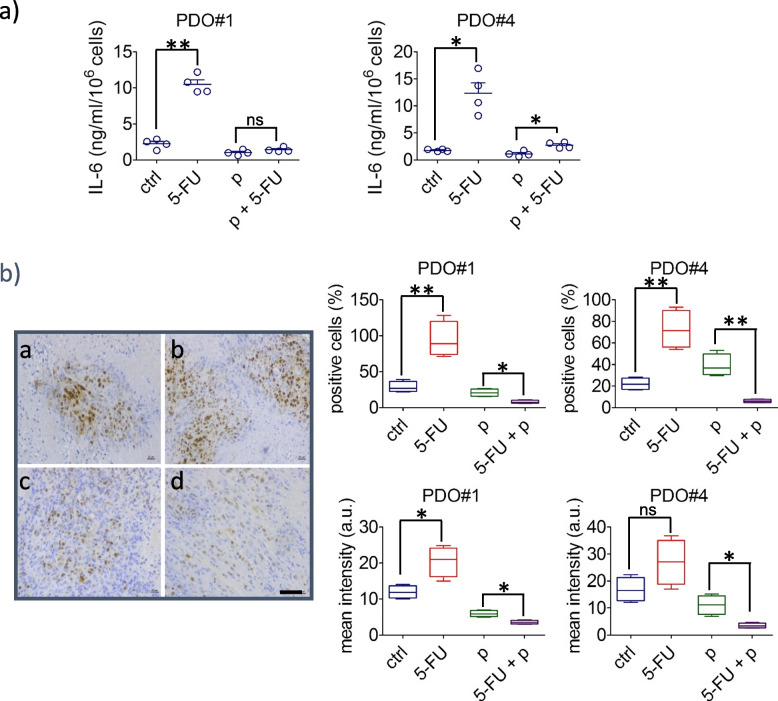
Treatment with pentoxifylline attenuated the release of IL-6 after 5-FU treatment. **a** Upper panel. Graph showing the levels of IL-6 quantified in the conditioned medium of the ctrl and 5-FU-treated PDOs. Results are expressed in nanograms per milliliter of IL-6, adjusted for 10^6 PDO-derived cells, after 48 h of medium conditioning and representative of three independent experiments. **b** Left panel: Representative immunocytochemistry of PDO#4-derived cells treated with ctrl (**a**), pentoxifylline (**b**), 5-FU (**c**) or pentoxifylline + 5-FU (**d**), cytospun and stained, after fixation, with an IL-6-antibody. Size bar, 100 µm. Right panel: Box plots showing the percentage of IL-6 positive cells (upper panels) and the mean intensity of the IL-6 signal (lower panel) in the PDO#1 and PDO#4 treated and stained as indicated in the left panel

Interleukin-6 (IL-6) may represent a pivotal factor mediating 5-FU resistance [39, 40]. IL-6 is known to promote the emergence of ALDHhigh cells in chemoresistant tumors [41–43]. Therefore, we tested whether this was the case for the 5-FU-treated CRC PDOs as well. This showed that pretreatment of the PDOs with a with an IL-6-neutralizing antibody (100 ng/ml, 1 h) before being challenged with ctrl- or 5-FU strongly blunted the 5-FU-elicited increase of the ALDHhigh cells in the CRC PDOs (Fig. 6c). This suggested that IL-6 may mediate the 5-FU stimulated increase of ALDHhigh cells in CRC PDOs. Further, to detail this observation, we evaluated the levels of IL-6 in ctrl, 5-FU. pentoxifylline (p) or pentoxifylline +5-FU treated PDOs, by both ELISA and ICC (Fig. 7a,b).

### Cancer Associated Fibroblasts (CAFs) highly contributed to the 5-FU elicited increase of IL-6

**Fig. 8:**
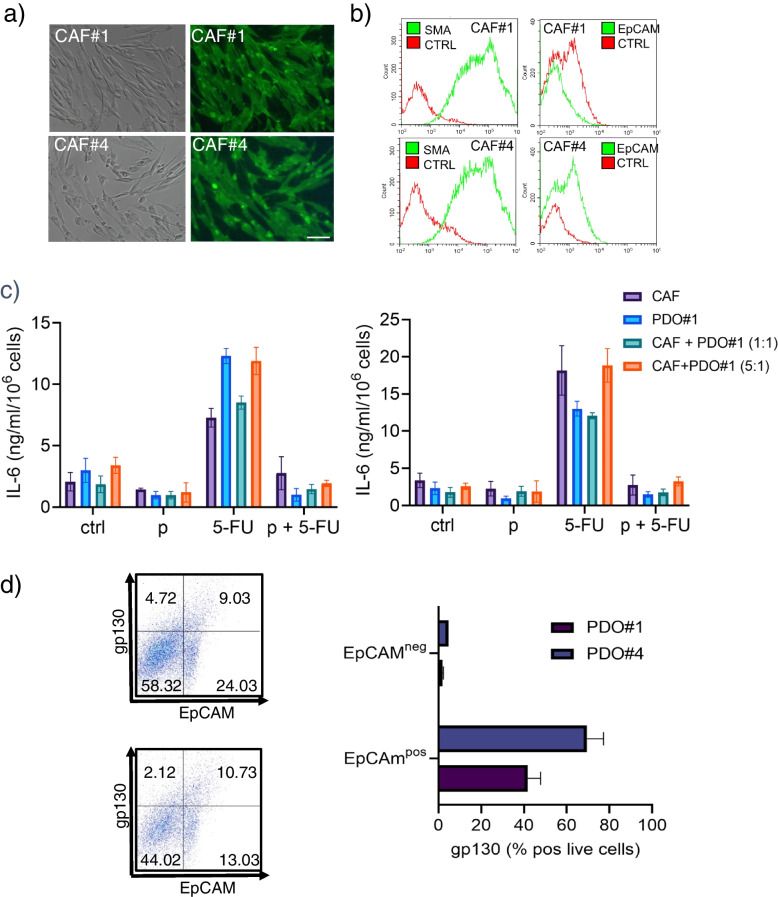
Cancer Associated Fibroblast are the main source of IL-6 after 5-FU treatment and may signal to gp130^pos^;EpCAM.^pos^ cells. CAFs were isolated as described in methods from the same specimens used for PDO generation and cocultured at different ratios with matched, early passage PDO#1 and PDO#4, to assess the levels of IL-6 released after ctrl, pentoxifylline, 5-FU or combined (pentoxifylline + 5-FU) treatment. **a** Representative micrographs of the CAF cultures derived from the same specimens of the PDO#1 (CAF#1) and PDO#4 (CAF#4). Left: bright field micrographs. Right: immunofluorescence staining with anti-SMA antibody. Size bar: 30 µm. **b** Overlay histogram plot of the CAF#1 and CAF#4 cultures stained with anti-SMA (Left) and with Anti-EpCAM (Right) antibodies and analyzed by flow cytometry. The background fluorescence from isotype-matched antibodies is also reported (**c**) Histograms showing the levels of IL-6 detected by an ELISA assay in the indicated samples. Results are expressed in nanograms per milliliter of IL-6, adjusted for 10^6 PDO-derived cells, after 48 h of medium conditioning and representative of three independent experiments. **d** Representative dot plots of the cocultures (1:1) stained with an anti-EpCAM antibody and with anti-gp130 antibody. Right panels. Quantitation of the results from the left panel for two independent experiments. Statistics: * *p* < 0.05; ** *p* < 0.01

Cancer associated fibroblasts (CAFs) are known to highly contribute to the chemotherapy response, in CRC and other cancer settings [44]. Further, remnant CAFs are still represented in early passage and lost in late passage PDOs [45] (and our observations). Therefore, we tested whether the increase in secreted IL-6 observed when treating PDOs with 5-FU could be ascribed to CAFs (Fig. 8). The identity of the isolated CAFs was verified by morphology, expression of SMA and absent expression of EpCAM (Fig. 8a,b). We evaluated the IL-6 levels in the conditioned medium of simultaneously derived CAFs, and early passage PDOs (≤ 2). PDO + CAFs were cultured at different ratios (1:1 and 1:5) (Fig. 8c). ELISA assay revealed that both CAF cultures secreted high amounts of IL-6, in a 5-FU- and pentoxifylline-sensitive way (Fig. 8c). An increased ratio between CAFs and PDO-derived cells strictly correlated with the increase of IL-6 (Fig. 8c), thus highlighting the CAFs as a source of IL-6.

### Gp130 expression in EpCAM positive cells may mediate the IL-6 signaling

Interleukin-6-induced signaling is initiated by binding of IL-6 to the IL-6 receptor and subsequent interaction with the signal transducing receptor subunit gp130. Binding of gp130 highly stabilizes the IL-6-IL6R complex and initiates signaling [46]. Thus, we investigated whether the released IL-6 could function in an autocrine or paracrine fashion through gp130. We explored this by co-staining the PDO + CAF culture -derived cells with EpCAM and gp130 antibodies and assessing by flow cytometry the percentage of positive cells within the treated PDOs (Fig. 8d). We found that gp130 was highly enriched within the EpCAM positive fraction but almost absent in the EpCAM negative fraction (Fig. 8d, left and right panel), which mostly includes remnant CAFs [28, 47]. This suggested a paracrine signaling increased by 5-FU and targeted to a gp130 receptor expressed on EpCAM positive cells within the pentoxifylline responsive PDOs.

### Pentoxifylline reduced the IL-6-driven STAT3(tyr705) phosphorylation

**Fig. 9:**
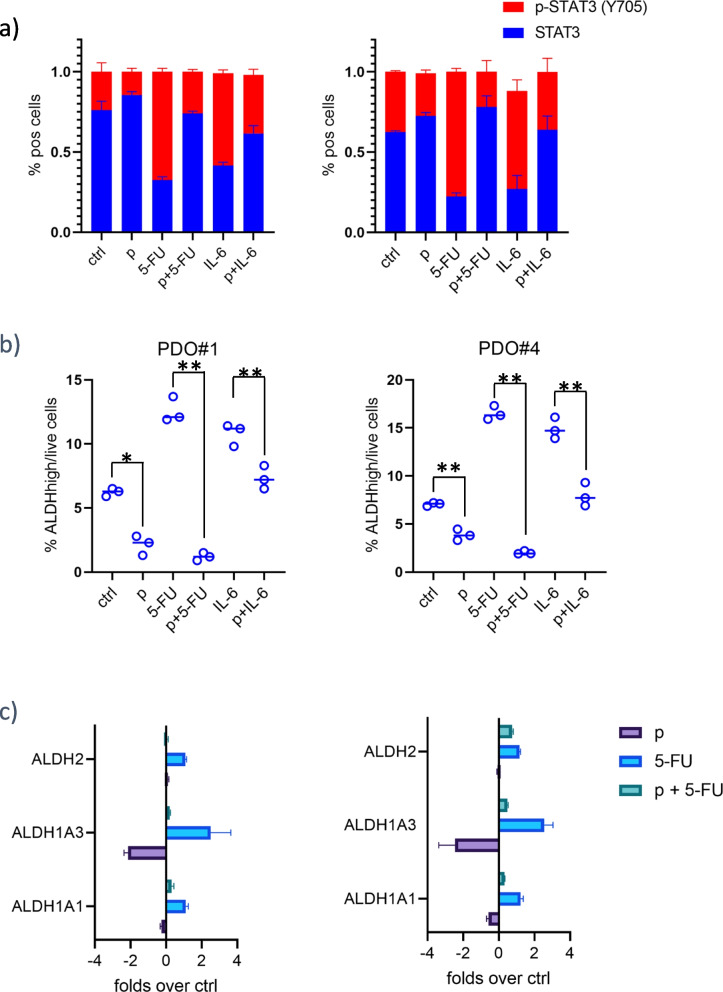
Pentoxifylline attenuated the IL-6 mediated increase of phosphorylated STAT3 in 5-FU-treated PDOs. **a** Graphs showing the levels of total and phosphorylated pSTAT3 (tyr705) detected by ELISA as indicated in the methods, from PDO#1 and PDO#4 treated with ctrl or 5-FU. Average of four independent experiments. **b**,**c** STAT3 inhibition by pentoxifylline affected the ALDHhigh cell number and the expression of ALDH1A3. Lower panels. Histograms reporting the percentage of ALDHhigh cells as assessed by flow cytometry in the same samples treated as in figure b, upper panels. Statistics: * *p* < 0.05; ** *p* < 0.01; no asterisk: not significant. **c** QRT-PCR was performed to assess the mRNA levels of he indicated ALDH isoforms in PDO#1 and PDO#4 cultures treated as shown for 24 h. Histograms show the folds above ctrl. The average of two independent experiments is reported. Statistics: * *p* < 0.05; ** *p* < 0.01

STAT3 is known to contribute to ALDHhigh cell homeostasis in various cancer settings [9, 19, 48] and STAT3 activation is a main signaling event after IL-6 stimulation of the cells [49]. Therefore, we evaluated the levels of phosphorylated STAT3 within the ctrl, pentoxifylline (p), 5-FU and p + 5-FU treated PDOs. Flow cytometry analysis revealed a strong increase of Tyr705-phosphorylated STAT3 elicited by 5-FU and readily blunted by pentoxifylline (Fig. 9a). This matched the changes in ALDHhigh cells within the treated PDOs (Fig. 9b), thus establishing a match between the attenuated STAT3 phosphorylation and the fluctuations seen in the ALDH high cells after treatment (Fig. 9a,b). Within the same experimental setting, we also evaluated whether exogenously added IL-6 could rescue the inhibitory effect of pentoxifylline on the STAT3 phosphorylation and on the ALDHhigh cell number (Fig. 9a,b). Addition of IL-6 (10 ng/ml) shortly (6 h) after pentoxifylline treatment partially rescued the effect of pentoxifylline on the STAT3 phosphorylation and on the percentage of ALDHhigh cells (Fig. 9a,b), validating the relevance of IL-6 release in the mechanism of action of pentoxifylline.

**Fig. 10:**
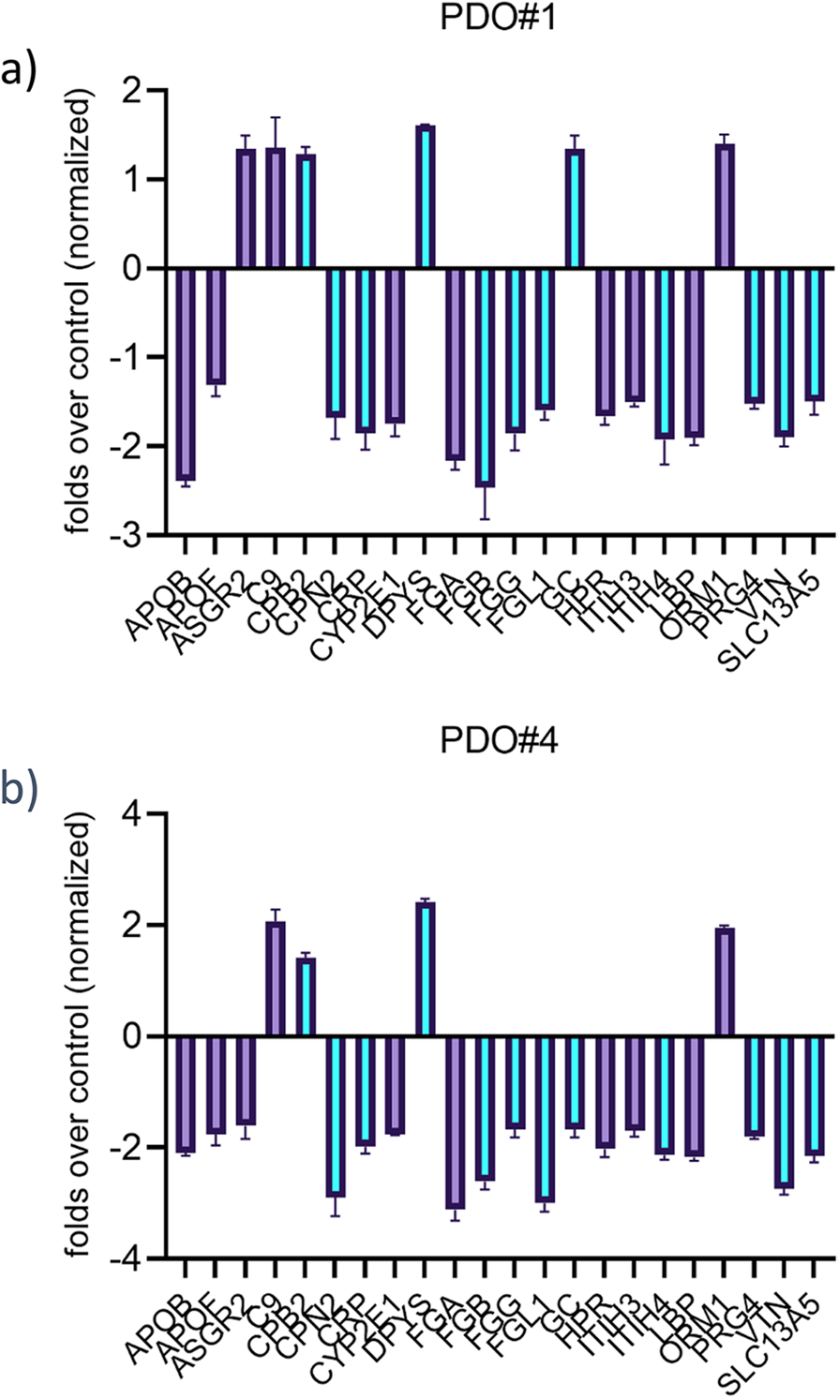
Pentoxifylline treatment partially reverted the 22-gene signature and downregulated most of the STAT3 putative target genes within the signature. Gene expression levels were assessed for the 22 genes comprising the identified colon to liver metastasis signature (Table 1) in pentoxifylline-treated PDO#1 and PDO#4 cultures (8 h, 20uM). Average of two independent experiments. Marked in purple are the genes deemed as or demonstrated to be STAT3 targets from literature data and promoter analysis

We also evaluated the effect of the pentoxifylline-based treatments on the mRNA levels of three ALDH isoforms, among which the ALDH1A3 is known as the most abundant in colon cancer tissues [50, 51]. This showed that pentoxifylline reduced the levels of ALDH1A3 mRNA in the PDO#1 and PDO#4 (when compared to ctrl-treated PDOs), while strongly attenuating the increase of ALDH1A3 elicited by 5-FU treatment (Fig. 9c). Altogether, this suggested that pentoxifylline may synergize with 5-FU by reducing the secreted IL-6 within the 5-FU treated CRC PDOs and thereby attenuating STAT3 signaling and the increase of chemoresistant ALDHhigh cells. Finally, we assessed the mRNA levels of the 22-gene signature originally used to identify pentoxifylline. Pentoxifylline treatment was administered in conditions (20uM, 8 h) roughly similar to those used to generate the CMAP data [52, 53]. This showed that pentoxifylline downregulated 16/22 genes and 18/22 genes, in PDO#1 and PDO#4, respectively (Fig. 10a,b). This partially validated the original CMAP targeted strategy, that the exposure of PDOs to pentoxifylline partially reversed the expression pattern of the biological signature. Further, pentoxifylline was capable of reducing the levels of most of the 22 genes, known to be STAT3 targets (https://maayanlab.cloud/Harmonizome/gene_set/STAT3/ENCODE+Transcription+Factor+Targets) implicating the STAT3 pathway engagement by this compound (Fig. 10).

## Discussion

We focused here on characterizing the molecular determinants of colon to liver metastasis and on identifying and validating druggable targets by means of a pharmacogenomic approach. Initially, we performed a detailed analysis of RNA sequencing data for a cohort of 50 patient-derived samples comprising primary CRC, liver metastasis and adjacent liver samples. From this analysis, two important observations were recorded. First of all, the number of differentially expressed (DE) genes between metastatic samples and unaffected adjacent liver tissue was lower when compared to DE genes between primary CRCs and adjacent liver tissue. Moreover, a large fraction of DE genes between primary CRCs and metastatic samples were characteristic of liver tissue, raising the intriguing possibility that metastatic cells may acquire features of the host tissue, while losing some of the characteristics of the primary lesion they originated from. This matches recent attempts to define a kinetic model of metastasis implying that the “normal stem cells” index in the host tissue may be related to the metastatic potential of the primary tumor [54]. Among the 42 genes expressed differentially between primary cancers and metastatic material, there is an absolute enrichment for liver-related genes and liver-related pathways. Further, of the 22 genes composing the "colon to liver" signature, none of them belongs to a classic EMTome or is being directly involved in migration, resistance to anoikis and evasion of immune surveillance. It is possible that the identified signature relates to a tissue adaptation ability endowed within the metastatic program. We speculate that overexpression of host tissue genes may represent a step required for completion of the metastatic process and thus, interfering with such a”host tissue” enriched signature may hold promise of attenuating organoid formation in the target organ. Thus, from this point of view, it may be worth evaluating drugs capable of interfering with the expression of a target-organ-enriched signature if this latter represents an important adaptive process instrumental to the metastasis survival. This is reminiscent of the “molecular mimicry with the host tissue”, a property evoked in the past for the metastatic cells. Still in line with this, we recently found that, adding host tissue- specific factors, greatly facilitated the propagation of metastatic breast cancer PDOs [55]. We also observed, when comparing frank metastatic lesions with matched primary CRCs, that the genomic difference between the primary tumor and metastatic material could be inadequate to explain the heterogeneity in disease progression [56]. Both of those latter experimental observations suggest a high dependency of the metastatic cells from signaling of the host, unaffected tissue, which goes beyond the genomic similarity with the primary tumor and may rely on host tissue factors.

Repurposing of clinically validated compounds is an effective way to find novel therapeutic approaches. It has potential for fast tracking into clinic due to the already characterized pharmacokinetic and safety profile of those drugs. In this work, we have employed CRC PDO cultures to partially validate the in silico results of the pharmaco-genomic screening. PDOs are interesting models which are amenable to clinically relevant observations since those may recapitulate the heterogeneity and cyto-architecture of the originating specimen [57]. In support of this, here we observed high correlation between the expression of CK20, EpCAM, Ki67 and CD44 in the CRC specimens and the obtained PDOs. Despite all the PDOs were derived from right colon adenocarcinoma specimens, we observed heterogeneity in their response to both 5-FU and to the experimental compounds here tested. We believe this provides support to the clinical usefulness of organoid based models to understand intra- and inter-tumor heterogeneity, a propelling force behind tumor progression.

We have found that three out of four CRC-PDOs were sensitive to pentoxifylline, administered as either single agent or co-administered with 5-FU. Pentoxifylline [3,7-Dimethyl-1-(5-oxohexyl)xanthine)], a xanthine family molecule, is a phosphodiesterase inhibitor, inflammatory cytokine regulator, immunomodulator, antioxidant and antifibrotic agent [58, 59]. It is FDA-approved for the symptomatic treatment of claudication. Pentoxifylline is reported to have anticancer activity [60], this observation dating back to 1991 [61] and has been considered as a sensitizer to chemotherapy and radiotherapy [62–64]. It was shown effective in combination with thiotepa, cisplatin, melphalan, doxorubicin, vincristine, mainly by increasing pro-apoptotic signaling [60]. In this report we observed synergy between 5-FU and pentoxifylline, consistent with a chemo sensitizing effect of the drug. In this experimental setting, pentoxifylline decreased the number of chemoresistant ALDHhigh cells by interfering with the IL-6 release after 5-FU treatment. IL-6 is a pivotal cytokine in many cancer settings, including CRC. In CRC, IL-6 levels are correlated with tumor stage, survival rate, and liver metastasis [13]. Reduced levels of IL-6 attenuated the STAT3 phosphorylation which in turn, strongly abated the number of chemoresistant ALDHhigh cells in the pentoxifylline + 5-FU treated PDOs. This also matches our observations on the relevance of STAT3 for the ALDH expression and survival of ALDHhigh cells [9, 10]. In line with the idea that high ALDH depicts a chemoresistant phenotype also in CRC, ALDH was shown as highly expressed in therapy-surviving tumors and in liver metastases [65]. Our proof of concept study suggest that pentoxifylline may have potential value of targeting CRC ALDHhigh cells in order to improve the efficacy of the standard treatment. Given the pleiotropic effects of pentoxifylline, it is likely that the IL-6-STAT3-ALDHhigh axis is not the only viable target of the drug. An effect of pentoxifylline on the intracellular GSH levels may represent an additional mechanism for chemosensitizing CRC cells. Pertinent to this, others and we have shown that engagement of the GSH pathway is a feature of chemoresistant breast cancer cell subpopulations [37, 66].

With regard to the other compounds, we note that dexketoprofen has potential anticancer actions, mainly through inhibition of cyclooxygenase 1 (COX-1) [67]. Notably, we did not observe synergism between dexketoprofen and 5-FU, in our experimental system. We did not collect here enough molecular data to support hypotheses explaining the lack of synergy: however, we may speculate that 5-FU treatment may stimulate pathways which antagonize the COX1 inhibition. It is known that chemotherapy elicits pro-inflammatory signaling [68], often linked to the onset of resistance [11]. On the other hand, since dexketoprofen is a selective COX1 inhibitor, it is possible that this latter enzyme may not be involved in the response to 5-FU.

A limit of this study is that we did not test combinatorial treatments among the four selected drugs. On one side, this may be relevant in light of the partial convergence in the mechanism of action of at least two of the compounds tested (e.g.: anti-inflammation), which may have led to potentiation mechanisms. Another limit of the present study is that we did not test the PDO response to the drug in the presence of tumor-microenvironment-associated components. We have provided evidence here that CAFs concurrently isolated and cocultured with the PDOs, exhibited a prominent ability to secrete IL-6 after 5-FU treatment, thus suggesting that CAFs may initiate a paracrine stimulation of the epithelial cancer cells, mediated by the expression of gp130, limited in our system to the EpCAm positive cells. On the other hand, the persistence of IL-6 secretion even in late passage PDOs (when CAFs are almost absent) may indicate that the CAF-mediated paracrine stimulation may be followed by autocrine production of IL-6 by the epithelial components. This strengthen the possibility of using pentoxifylline as an adjuvant in in vivo settings, were CAFs and many other TME components are not artefactually separated or reduced by PDO passaging. It also raises the suggestion of using early passage PDOs for assessing the contribution of TME remnants to the drug response, when coculturing is not feasible. A systematic coculturing approach is ongoing and will be addressed in future studies on a larger cohort of CRC-PDOs.

## Conclusions

This proof of concept study shows that PDOs represent a suitable platform to validate molecular data, to test clinically viable compounds and, ultimately, obtain key insights on the mechanism of action of the identified drug candidates. Pentoxifylline may represent an interesting candidate for combinatorial therapy. A larger cohort of samples will be needed to further validate those observations.

## Supplementary Information

**Additional file 1.**

**Additional file 2.**

## Data Availability

All data generated or analyzed during this study are included in this published article (and its supplementary information files).

## Abbreviations

CRC: ColoRectalCancer
5-FU 5: Fluorouracile
ALDH: Aldehyde dehydrogenase
PDO: Patient-Derived-Organoid
IL-6: Interleukin-6
IL-10: Interleukin-10
TNF-alpha: Tumor Necrosis Factor alpha
CK20: Cytokeratin-20
EpCAM: Epithelial cell adhesion molecule
RAS: Rat sarcoma
STAT3: Signal transducer and activator of transcription 3
ZEB1: Zinc finger E-box-binding homeobox 1
CD73: Cluster of differentiation 73
EPS: Encapsulating peritoneal sclerosis
EMT: Epithelial to mesenchymal transition
COX: Cyclooxygenase
VAF: Variant allele frequency
GP130: Glycoprotein 130
TME: Tumor-Micro-Environment
EGFR: Epidermal Growth Factor Receptor
