# Supplementary material for: Interrogating colorectal cancer metastasis to liver: a search for clinically viable compounds and mechanistic insights in colorectal cancer Patient Derived Organoids
Source: J Exp Clin Cancer Res. 2023 Jul 17;42:170. doi: 10.1186/s13046-023-02754-6 (PMC10351152; doi:10.1186/s13046-023-02754-6)
Supplement: Supplementary file 1 — Additional file 1. [file 13046_2023_2754_MOESM1_ESM.pptx]

## Slide 1
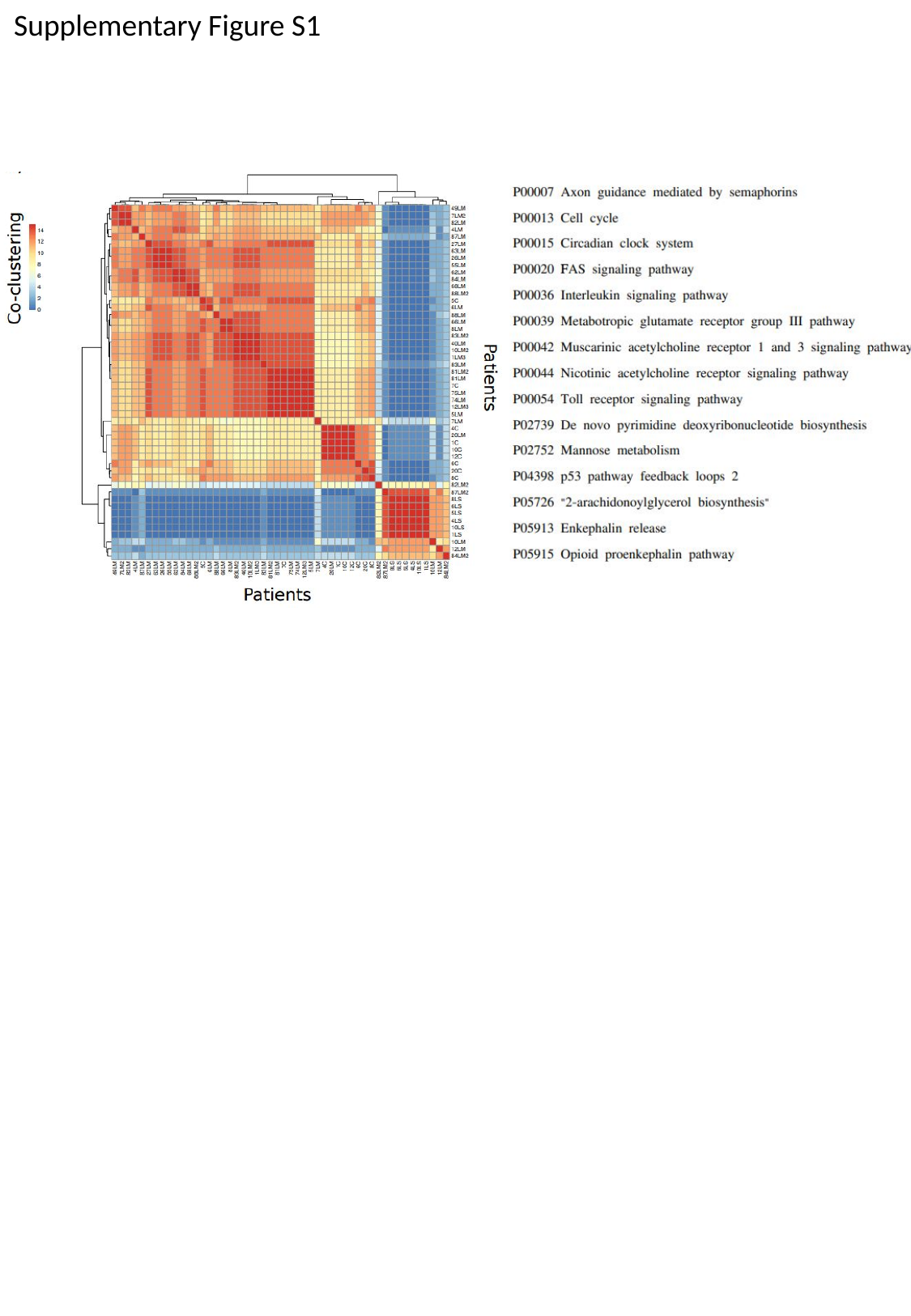

Supplementary Figure S1

## Slide 2
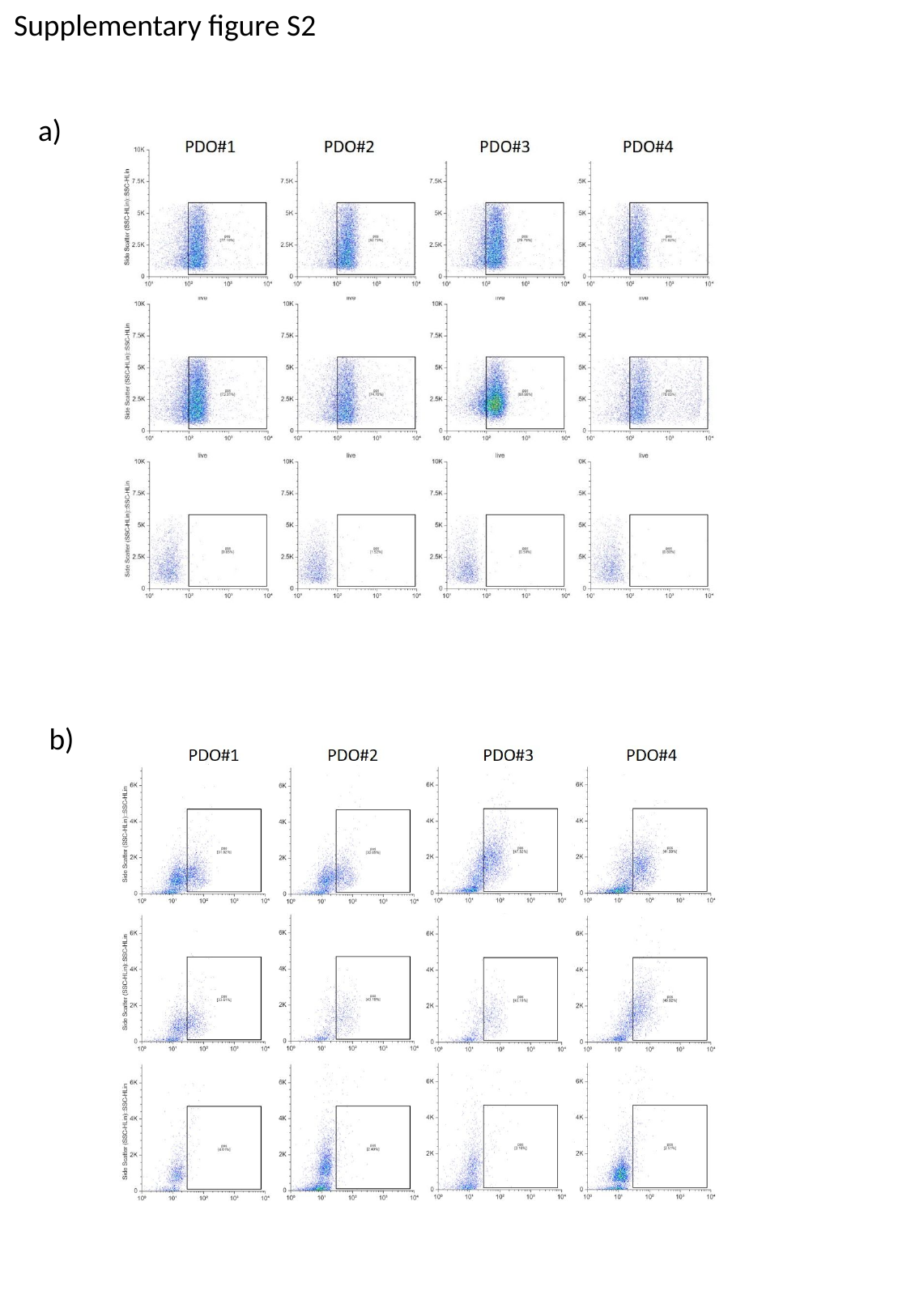

Supplementary figure S2
a)
b)

## Slide 3
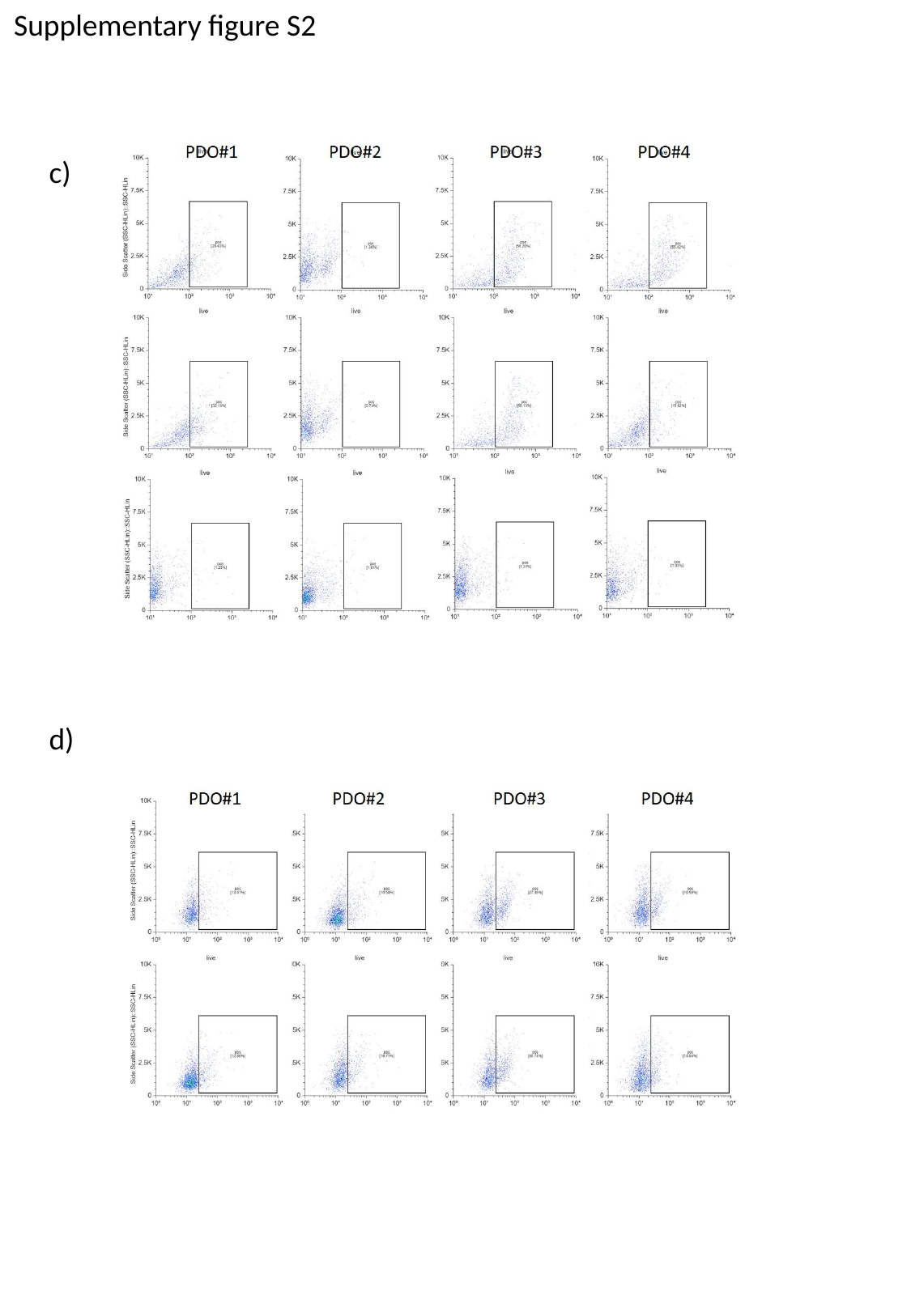

Supplementary figure S2
c)
d)

## Slide 4
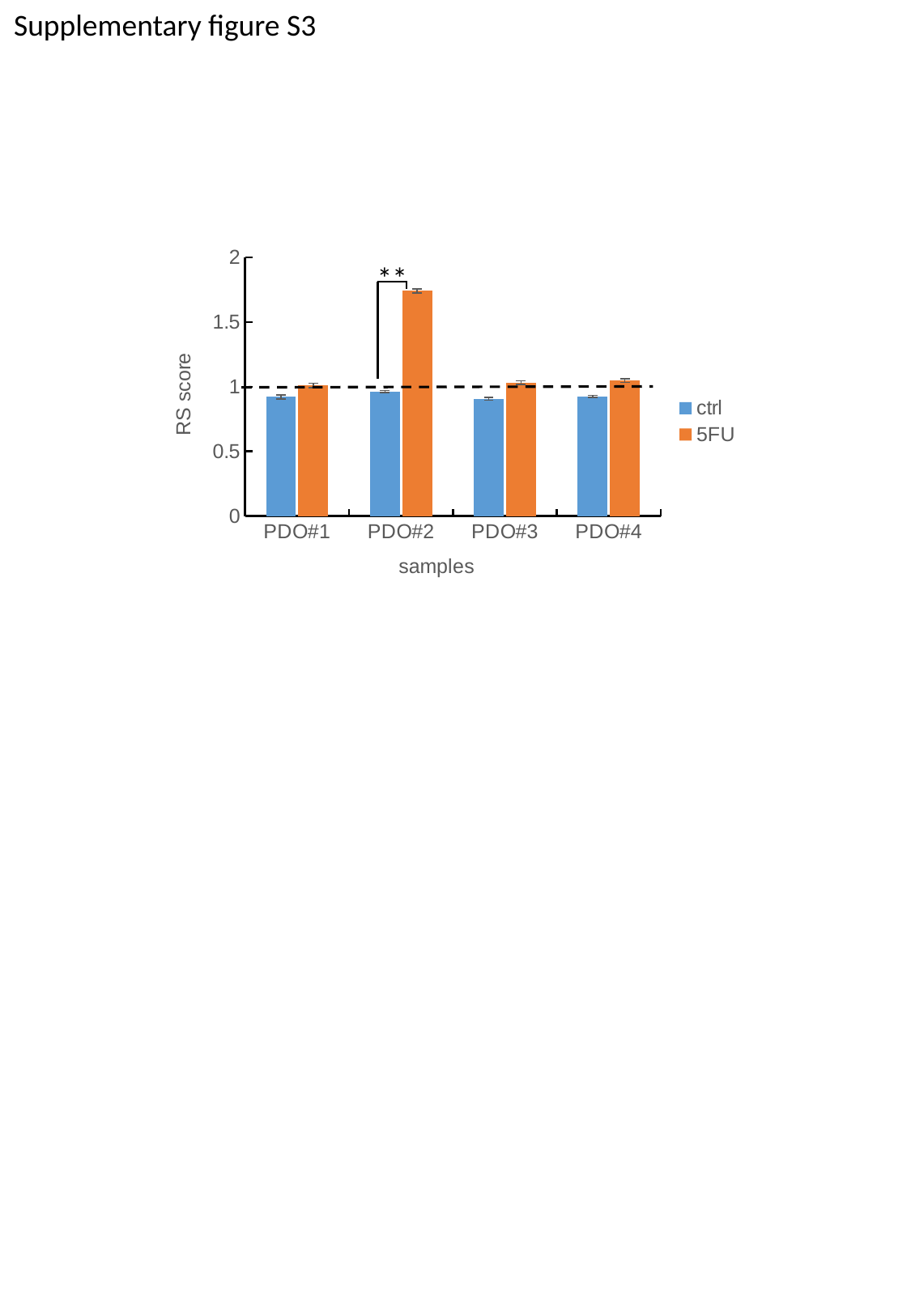

Supplementary figure S3
### Chart
| Category | ctrl | 5FU |
|---|---|---|
| PDO#1 | 0.921582935 | 1.010295 |
| PDO#2 | 0.9632298593434999 | 1.740799525 |
| PDO#3 | 0.90726475035 | 1.0325817 |
| PDO#4 | 0.92635083146835 | 1.0467514976999999 |**
